# Ethnic variation in stillbirth risk and the role of maternal obesity: analysis of routine data from a London maternity unit

**DOI:** 10.1186/s12884-014-0404-0

**Published:** 2014-12-07

**Authors:** Nicole Penn, Eugene Oteng-Ntim, Laura L Oakley, Pat Doyle

**Affiliations:** King’s College London School of Medicine, Capital House, King’s College, London, SE1 3QD UK; Guy’s and St Thomas’ NHS Foundation Trust, Westminster Bridge Road, London, SE1 7EH UK; Department of Non-communicable Disease Epidemiology, London School of Hygiene and Tropical Medicine, Keppel Street, London, WC1E 7HT UK

**Keywords:** Stillbirth, Obesity, Ethnicity

## Abstract

**Background:**

Approximately 5 in 1,000 deliveries in England and Wales result in stillbirth, with little improvement in figures over the last few decades. The aim of this study was to investigate the association between clinical and socio-demographic factors and stillbirth, with a particular focus on ethnicity and obesity.

**Methods:**

Analysis of routine maternity data on 53,293 singleton births occurring in a large London teaching hospital between 2004 and 2012. Logistic regression was used to investigate risk factors for stillbirth and to explore potential effect modification.

**Results:**

53,293 deliveries occurred during the time period, of which 329 resulted in a stillbirth (6.2 per 1,000 births). Compared to White women, non-White ethnicity was associated with a doubling of the odds of stillbirth (aOR for Black women 2.15, 95% CI 1.56-2.97; aOR for South Asian women 2.33, 95% CI 1.42-3.83). Obese women had a trend towards higher odds of stillbirth compared to women of recommended BMI (aOR 1.38, 95% CI 0.98-1.96), though this was not significant (p 0.07). Both higher parity (≥2 compared to para 1) and hypertension were associated with a higher odds of stillbirth (parity ≥2 aOR 1.65, 95% CI 1.13-2.39; hypertension aOR 1.84, 95% CI 1.22-2.78) but there was no evidence that area deprivation or maternal age were independently associated with stillbirth in this population. There was some evidence of effect modification between ethnicity and obesity (p value for interaction 0.06), with obesity a particularly strong risk factor for stillbirth in South Asian women (aOR 4.64, 95% CI 1.84-11.70).

**Conclusions:**

There was a high prevalence of stillbirth in this multi-ethnic urban population. The increased risk of stillbirth observed in non-White women remains after adjusting for other factors. Our finding of possible effect modification between ethnicity and obesity suggests that further research should be conducted in order to improve understanding of the interplay between ethnicity, obesity and stillbirth.

**Electronic supplementary material:**

The online version of this article (doi:10.1186/s12884-014-0404-0) contains supplementary material, which is available to authorized users.

## Background

In 2012 the prevalence of stillbirth in England and Wales was 4.9 stillbirths per 1,000 total births [1], with an overall decline in prevalence over the preceding decade [2]. However, there is little evidence of improvement in stillbirth rates among singleton pregnancies during this time [3].

Identifying the cause of stillbirth can be challenging due to the plurality of classification systems, many of which result in a high proportion of cases remaining unexplained [4]. Common causes in UK studies are congenital anomaly, antepartum haemorrhage, maternal medical conditions and infection [5,6]. Meta-analysis of studies conducted in high-income countries has identified the following risk factors for stillbirth; overweight and obesity, pre-existing diabetes and pre-existing hypertension, advanced maternal age, primiparity, illicit drug use, low education and low socioeconomic status, no antenatal care, pregnancy-related hypertension, small for gestational age, post-term pregnancy, and previous stillbirth [7]. Many of these associations have been confirmed in UK studies [6,8-10].

The UK 2009 Perinatal Mortality report found Black women nationally to have twice the odds of stillbirth compared to White women, while Asian women had a smaller but significant increase of 60% in their odds of stillbirth compared to White women [2]. Nationally women of these ethnicities account for only a small proportion of births. However, 2009 estimates suggest that 40.5% of London’s population is from a non-White British background [11], with this proportion likely to be higher among the maternal population due to the younger age structure of non-White ethnic groups. Previous studies have reported that among women living in London, Black and Asian women have a higher risk of stillbirth compared to White women [6,12]. In the UK, Black women have higher rates of obesity in pregnancy compared to White women [13]. Little is known about the role of obesity in explaining the higher risk of stillbirth observed in Black and South Asian women in the UK, although there is evidence from other high income countries that the association between obesity and obstetric outcomes varies by ethnicity [14,15]. These studies, predominately conducted in the US, may not be generalizable to the UK due to differences in the composition of ethnic groups between the two countries.

The aim of this study was to investigate the association between clinical and socio-demographic factors and stillbirth in an urban UK population, with a particular focus on the role of ethnicity and obesity. The study population, from south London, is ethnically diverse and has relatively high levels of deprivation and maternal obesity compared to the UK as a whole.

## Methods

Data were extracted from a routine database kept on all patients delivering at Guy’s and St Thomas’s Hospital Foundation Trust (GSTT). The hospitals managed by this Trust are located in south London, UK. Direct patient identifiers were removed from the dataset before analysis and ethical approval was granted by the London School of Hygiene and Tropical Medicine Ethics committee. All 53,293 deliveries between January 2004 and May 2012 which ended in a singleton livebirth or stillbirth were included in this analysis.

### Outcome

The primary outcome was stillbirth, defined as ‘a baby delivered with no signs of life known to have died after 24 completed weeks of pregnancy’ [16]. For the purposes of international comparison, a the number of stillbirths according to the WHO recommended threshold of 28 weeks is presented as an (Additional file 1: Table S1).

### Explanatory variables

Ethnicity was classified as White (British, Irish, White Other), Black (Black African, Black Caribbean, Other Black), South Asian (Indian, Pakistani, Bangladeshi, Asian Other), or Other (Chinese, Mixed, Other). Maternal age was grouped as <20 years, 20–24 years, 25–29 years, 30–34 years, 35–39 years and 40+ years for descriptive tables and modelled as a continuous variable for regression modelling.

Deprivation was measured by the English Indices of Multiple Deprivation (IMD); a recognised scoring system for area-based deprivation based on census data covering seven different domains [17]. The IMD (2007) score was calculated using postcode at antenatal care booking, and scores were mapped to national IMD quintiles with quintile 1 as the least deprived and quintile 5 as the most deprived.

Maternal body mass index (BMI) was calculated from the mother’s height and weight recorded at booking (weight in kg/(height in m)^2^) and categorised as ‘underweight’ (<18.5), ‘recommended’ (18.5-24.9), ‘overweight’ (25–29.9), or ‘obese’ (30+); implausible values (<13, >70) were coded as missing. We created a second BMI variable for use in sensitivity analyses, applying lower thresholds for South Asian women only (overweight ≥23, obese ≥27.5) as discussed in recent NICE guidance [18].

Women were considered to be a ‘hypertension’ case when any pre-existing or pregnancy related hypertension was recorded, and a ‘diabetes’ case when any pre-existing type 1 or 2 or gestational diabetes was recorded. This information was recorded prospectively i.e. before delivery. Parity was categorised into three groups (nulliparous – para 0, para 1, or para 2+), with para 1 as the reference group to reflect the increased risk of stillbirth in primiparous women [7]. Additional explanatory variables were marital status (single vs. in a relationship), and smoking status at booking (yes/no).

### Statistical analysis

We calculated the prevalence of stillbirth as the number of singleton stillbirths divided by the total number of births after 24 completed weeks, presented per 1,000 total singleton births. Odds ratios for the association between explanatory factors and stillbirth were calculated using univariate and multivariable logistic regression. Obesity and ethnic group were considered the main explanatory factors and were included in all multivariable models, other variables were included in multivariable modelling where there was evidence that they were independently associated with stillbirth (p < 0.05 for at least one parameter using the Wald test after adjustment for other variables). Potential effect modification between ethnicity and BMI, parity and area deprivation was explored using an interaction term in the final logistic regression model. Interaction terms assessed as p < 0.05 using the Wald test were considered significant; and p values <0.1 assessed as providing possible evidence of interaction.

To account for the fact that some women contributed more than one birth to the analysis, all confidence intervals were calculated using robust standard errors.

Where data were missing for >10% observations for a variable included in multivariable analysis, a sensitivity analysis was run using the final model fitting a dummy category for missing data to ensure similar results were obtained using the full sample. Additionally, to address the debate surrounding the use of conventional BMI thresholds to assess risk of chronic health conditions in South Asian populations [18], the final multivariable model was repeated using lower BMI thresholds for South Asian women. The results of this model were compared to the main analysis.

All statistical analysis was conducted using Stata version 13.

## Results

### Prevalence of stillbirth

Of the 53,293 singleton births in this analysis, 329 resulted in stillbirth: an overall rate of 6.2 stillbirths per 1,000 births (95% CI 5.5-6.9) (Table 1). There was slight variation in the stillbirth rate by year of delivery, with the highest rate mid-period in 2008/09. The prevalence of stillbirth was higher in all non-White groups compared to White women: 9.1 per 1,000 for both South Asian and Black women, 6.9 for other ethnic groups, and 3.8 for White women (Additional file 2: Table S2).

**Table 1:** **Crude stillbirth rate by year of delivery (n = 53,293)**

		**Total births**		**Stillbirths**		**Stillbirth rate (per 1,000 total births)**	**Crude OR (95% CI)**	**p value**
		**n**	**(%)**	**n**	**(%)**			
**All births**		53,293		329		6.2		
**Year of delivery**	2004/05	11,361	(21.3)	70	(21.3)	6.2	ref	
	2006/07	12,347	(23.2)	79	(24.0)	6.4	1.04 (0.75-1.43)	0.82
	2008/09	13,241	(24.8)	96	(29.2)	7.3	1.18 (0.86-1.61)	0.30
	2010/11/12^1^	16,344	(30.7)	84	(25.5)	5.1	0.83 (0.60-1.15)	0.26

^1^6 months of data for 2012 combined with 2010/11.

For the 202 stillbirths where results of the initial exam were reported, nearly two-thirds (64%, n = 129) were known to be antepartum deaths due to a reported macerated appearance. A further 36% (n = 73) of stillbirths were reported as fresh intrauterine deaths however it cannot be known how many of those were the consequence of an antepartum rather than intrapartum cause. The majority (62.0% n = 204) of stillbirths occurred before 37 weeks gestation and only 10% were delivered after 40 weeks gestation.

### Characteristics of the sample

Table 2 describes the characteristics of the mothers in the sample. In summary, 59% of mothers were aged 30 and over at the time of delivery, the majority of mothers were either White (49.5%) or Black (32.5%), and nearly four-fifths (79%) lived in the two most deprived IMD quintiles nationally. More than 30% of mothers were overweight or obese and 58% were nulliparous. One quarter (26.6%) of all women were single. Key characteristics stratified by ethnicity are presented as an (Additional file 1: Table S1).

**Table 2:** **Univariable analysis of clinical and socio-demographic factors associated with stillbirth (n = 53,293)**

		**Total births**		**Stillbirths**		**Crude OR (95% CI)**	**p value**
		**n = 53293**	**(%)**	**n = 329**	**(%)**		
**Ethnicity**	White	26,390	(49.5)	99	(30.1)	ref	
	Black	17,337	(32.5)	157	(47.7)	2.43 (1.88-3.13)	<0.001
	Asian	2,957	(5.5)	27	(8.2)	2.45 (1.60-3.75)	<0.001
	Other	5,790	(10.9)	40	(12.2)	1.85 (1.27-2.69)	0.001
	*Missing*	*813*	*(1.5)*	6	*(1.8)*		
**Deprivation (IMD)**	Quintile 1 (least deprived)	1,636	(3.1)	7	(2.1)	0.64 (0.30-1.37)	0.25
	Quintile 2	3,056	(5.7)	16	(4.9)	0.78 (0.46-1.32)	0.36
	Quintile 3	6,072	(11.4)	31	(9.4)	0.76 (0.51-1.14)	0.19
	Quintile 4	23,997	(45.0)	151	(45.9)	0.94 (0.74-1.20)	0.64
	Quintile 5 (most deprived)	18,147	(34.1)	121	(36.8)	ref	
	*Missing*	*385*	*(0.7)*	3	*(0.9)*		
**Maternal age**	<20 yrs	2,041	(3.8)	11	(3.3)	0.84 (0.44-1.58)	0.58
	20-24 yrs	7,217	(13.5)	39	(11.9)	0.84 (0.56-1.24)	0.38
	25-29 yrs	12,125	(22.8)	78	(23.7)	ref	
	30-34 yrs	17,473	(32.8)	100	(30.4)	0.89 (0.65-1.20)	0.45
	35-39 yrs	11,441	(21.5)	74	(22.5)	1.00 (0.73-1.39)	0.97
	40+ yrs	2,996	(5.6)	27	(8.2)	1.40 (0.90-2.18)	0.13
	Per year increase in age					*1.02 (1.00-1.04)*	*0.039*
**BMI**	Underweight	1,413	(2.7)	7	(2.1)	1.01 (0.47-2.16)	0.98
	Recommended	24,423	(45.8)	120	(36.5)	ref	
	Overweight	10,503	(19.7)	70	(21.3)	1.36 (1.00-1.84)	0.046
	Obese	6,339	(11.9)	61	(18.5)	1.97 (1.44-2.68)	<0.001
	*Missing*	*10,615*	*(19.9)*	*71*	*(21.6)*	*1.36 (1.02-1.83)*	*0.04*
**Parity**	Nulliparous	30,856	(57.9)	168	(51.1)	1.03 (0.76-1.36)	0.85
	Para 1	13,196	(24.8)	70	(21.3)	ref	
	Para 2+	9,170	(17.2)		(27.7)	1.88 (1.37-2.57)	<0.001
	*Missing*	*71*	*(0.1)*	*0*	*(0.0)*		
**Single status**	Married or living with partner	38,987	(73.2)	240	(72.9)	ref	
	Single	14,167	(26.6)	88	(26.7)	1.01 (0.79-1.29)	0.94
	*Missing*	*139*	*(0.3)*	1	*(0.3)*		
**Smoking**	Non-smoker at booking	44,205		278	(84.5)	ref	
	Smoker at booking	3,327		26	(7.9)	1.01 (0.79-1.29)	0.29
	*Missing*	*5,761*	*(10.8)*	*25*	*(7.6)*		
**Hypertensive disorders**	No hypertension	50,721	(95.2)	295	(89.7)	ref	
	Hypertension	2,572	(4.8)	34	(10.3)	2.29 (1.61-3.26)	<0.001
**Diabetes**	No diabetes	52,064	(97.7)	317	(96.4)	ref	
	Diabetes	1,229	(2.3)	12	(3.6)	1.61 (0.90-2.87)	0.11

For both smoking status and BMI there were considerable levels of missing data (11% and 20% respectively); for all other variables less than 2% of records were missing data.

### Univariate analysis

The results of univariate logistic regression are presented in Table 2. Maternal age (modelled as a continuous variable) was significantly associated with stillbirth (OR 1.02 per unit increase in age, 95% CI 1.00-1.04, p 0.039), with the highest odds of stillbirth occurring in women ≥40 years. Area deprivation, as measured by IMD, did not appear to be associated with stillbirth in this crude analysis, nor did single status or smoking status. Women with two or more previous children had a higher odds of stillbirth compared to women with only one previous child and women of all ethnicities had higher odds of stillbirth than White women. Both overweight women and obese women had a higher odds of stillbirth compared to women of recommended BMI (OR for overweight women: 1.36, 95% CI 1.00-1.86, p = 0.046; obese women: OR 1.97, 95% CI 1.44-2.68, P < 0.001).

### Multivariable analysis

Ethnicity remained strongly associated with stillbirth in multivariable analysis (Table 3). Using White women as the comparison group, Black women had twice the odds of stillbirth (aOR 2.15, 95% CI 1.56-2.97, p < 0.001) and South Asian women 2.3 times the odds (aOR 2.33, 95% CI 1.42-3.83, p < 0.001).

**Table 3:** **Multivariable analysis of clinical and socio-demographic factors associated with stillbirth (n = 42,043)**

		**Adjusted OR** ^**1**^ **(95% CI)**	**p value**
**Ethnicity**	White	ref	
	Black	2.15 (1.56-2.97)	<0.001
	Asian	2.33 (1.42-3.83)	0.001
	Other	1.99 (1.32-3.01)	0.001
**Maternal age**	*per year increase in age*	1.03 (1.01-1.06)	0.012
**BMI**	Underweight	1.04 (0.49-2.23)	0.911
	Recommended	ref	
	Overweight	1.03 (0.75-1.42)	0.847
	Overweight	1.38 (0.98-1.96)	0.068
**Parity**	Nulliparous	1.28 (0.92-1.79)	0.137
	Para 1	ref	
	Para 2+	1.65 (1.13-2.39)	0.009
**Hypertensive disorders**	No hypertension	ref	
	Hypertension	1.84 (1.22-2.78)	0.004

^1^Odds ratios adjusted for all other variables in table.

The dose response association between maternal BMI and stillbirth observed in univariate analysis was somewhat attenuated after adjustment, with no evidence of increased odds of stillbirth among overweight women (aOR 1.03, 95% CI 0.75-1.42, p 0.85), and the OR for obese women just over the p 0.05 significance level (aOR 1.38, 95% CI 0.98-1.96, p 0.07).

After adjustment, women with two or more previous births had a higher odds of stillbirth (aOR 1.65, 95% CI 1.13-2.39, p 0.009) compared to women with only one previous birth. Hypertension was associated with a near doubling of the odds of stillbirth (aOR 1.84, 95% CI 1.22-2.78, p 0.004).

The final logistic regression model was run on an expanded sample including observations with missing data on BMI, fitting a dummy category for ‘BMI – missing’. The results of this analysis were not notably different from the analysis using the restricted sample (Additional file 3: Table S3).

### Effect modification

**Figure 1:**
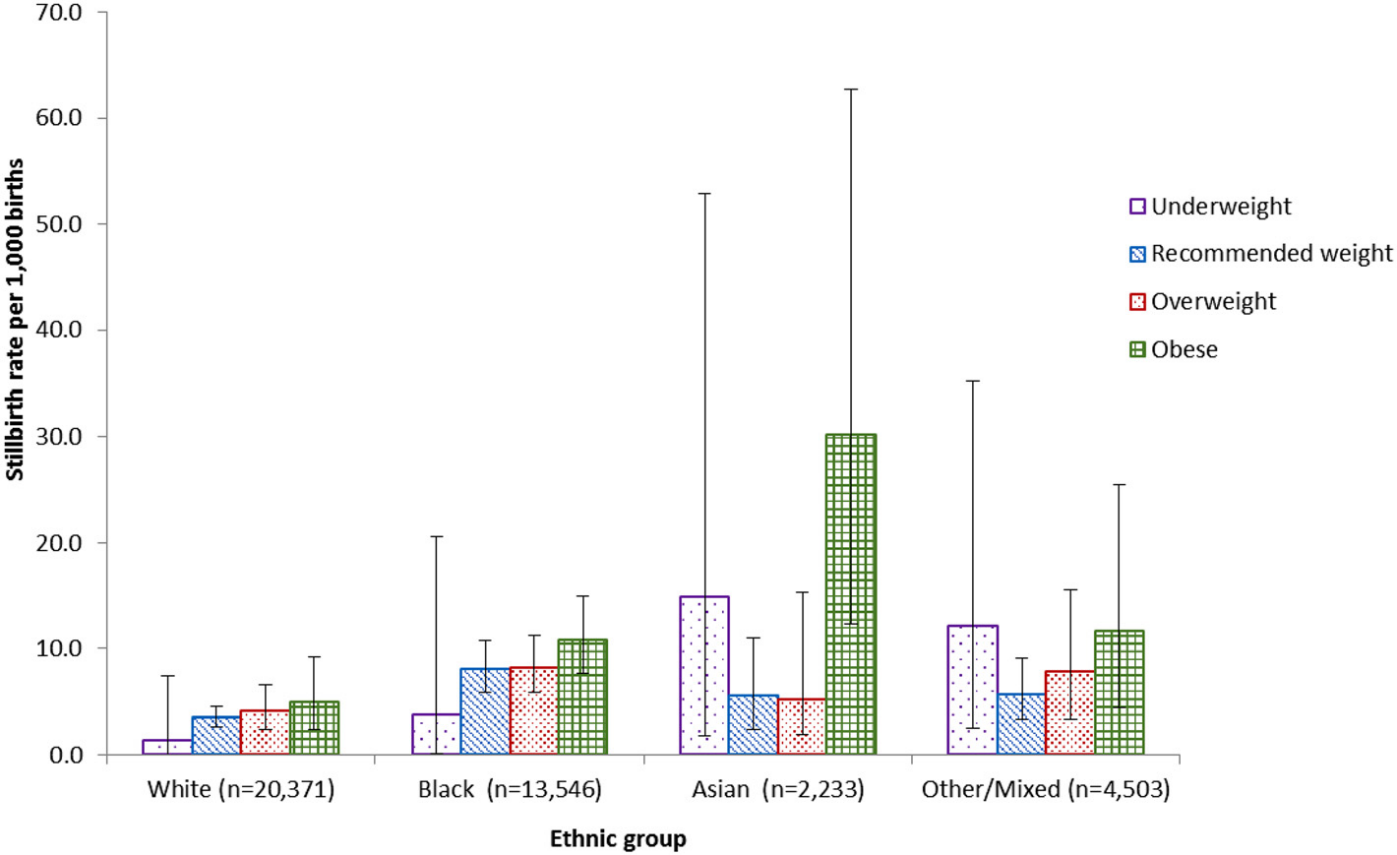
**Stillbirth rate by maternal BMI category and ethnic group.**

There was no evidence of interaction between ethnicity and either parity or deprivation in either stratified univariable analysis or multivariable analysis. However, stratified analysis suggested potential effect modification between ethnicity and maternal BMI. Among White and Black women, there was a consistent dose response relationship between increasing BMI from the recommended weight category onwards (recommended, overweight, and obese) and stillbirth rate. However, the relationship between BMI and stillbirth risk was exceptional among South Asian women with a disproportionately high stillbirth rate observed among obese women (Figure 1). To further investigate the possibility of effect modification an interaction term was included in the adjusted regression model with maternal BMI modelled as a binary variable (obese vs. not obese, underweight women included in the reference category) in order to maximise power. The Wald statistic for the interaction term provided some evidence (p = 0.05) of effect modification. Stratified adjusted odds ratios (Table 4) demonstrate that obesity is a strong independent risk factor for stillbirth among South Asian women, though there was little evidence that obesity was associated with stillbirth in other ethnic groups.

**Table 4:** **Adjusted odds ratios for the association between obesity and stillbirth, stratified by ethnic group**

		**Births**	**Stillbirths**	**Stillbirth rate (per 1,000 total births)**	**Adjusted OR** ^1^ **(95% CI)**	**p value**
		**n**	**n**			
**White**	Not obese	19116	68	3.6	ref	
	Obese	2032	10	4.9	1.32 (0.68-2.57)	0.41
**Black**	Not obese	10326	83	8.0	ref	
	Obese	3503	38	10.8	1.14 (0.76-1.71)	0.52
**Asian**	Not obese	2137	13	6.1	ref	
	Obese	232	7	30.2	4.64 (1.84-11.70)	0.001
**Other**	Not obese	4240	28	6.6	ref	
	Obese	513	6	11.7	1.60 (0.68-3.78)	0.28

^1^Adjusted for maternal age, hypertension and parity.

### South Asian specific BMI thresholds

Applying lower thresholds for overweight and obesity in South Asian women resulted in a higher proportion of South Asian women being classified as overweight or obese (52.4% vs. 34.1% using conventional thresholds). In multivariable analysis (Additional file 4: Table S4a) the odds ratio for the association between South Asian ethnicity and stillbirth was very slightly attenuated by using lower BMI thresholds to define overweight and obesity (aOR for South Asian women using alternative BMI thresholds 2.26, 95% 1.37-3.72; aOR using conventional thresholds 2.33, 95% 1.42-3.83). In stratified analysis (Additional file 4: Table S4b), the OR for the association between obesity and stillbirth in South Asian women was considerably lower using alternative BMI thresholds (aOR 2.83, 95% CI 1.17-6.85 vs. aOR 4.64, 95% CI 1.84-11.70).

## Discussion

### Main findings

In this study conducted using a diverse urban population, a number of maternal clinical and socio-demographic factors were independently associated with stillbirth. In particular, non-White ethnicity was significantly associated with stillbirth after adjustment for factors, and there was evidence to suggest that the association between obesity and stillbirth varied by ethnic group. A notable observation was the high risk of stillbirth in obese South Asian women.

### Strengths and limitations

This study was conducted using a unique maternal population: urban, multi-ethnic and with a high degree of deprivation. A large number of variables were available for analysis, although the information was not available (or incomplete) for some variables of interest, for example likely timing of fetal death (antepartum or intrapartum), classification of stillbirth type (underlying cause and associated factors), previous complications, fetal growth restriction, and congenital anomalies. In particular, fetal growth restriction and congenital anomalies have been hypothesised as variables key in explaining the increased risk of stillbirth in non-White women. For most included variables there was a low level of missing data. However, for BMI, approximately 1 in 5 mothers had missing data and these women were not included in final adjusted models. This is a significant limitation of the analysis. Although there was little evidence that women with missing BMI differed with respect to any key characteristics, the group with missing BMI tended to be slightly older, less deprived, less likely to have diabetes, and more likely to be nulliparous (data not shown). All these characteristics are associated with a lower prevalence of obesity, suggesting that women with missing BMI may be more likely to be non-obese (i.e. data are “missing not at random”). This is clinically plausible, as BMI may be more likely to be measured by clinicians where women are visibly overweight. Sensitivity analysis including a dummy category for ‘BMI – missing’ showed that the estimates were highly similar when the women with missing BMI were included in the models. The fact that women with missing BMI status appear to have an increased odds of stillbirth in univariate analysis suggests that these women may have other risk indicators which were unmeasured in our analysis.

The relative rarity of stillbirth in this population means estimates in sub-group analysis have in some cases been calculated from a small number of events, resulting in wide confidence intervals. The analysis was likely underpowered to examine interaction, with only 27 stillbirths in one of the key groups of interest (South Asian women) group. In addition, under-reporting of hypertensive and diabetic conditions may have led to residual confounding by these factors in the adjusted analysis. Information on whether diabetes was pre-existing or gestational was not available for all diabetes cases, so we chose to combine these two conditions. This may have had the effect of underestimating the stillbirth risk associated with pre-existing diabetes as there is little evidence that gestational diabetes is associated with stillbirth [8].

Ethnicity was self-reported by mothers and recorded by the midwife at booking. For the analysis reported here, ethnicity was combined into four categories (White, South Asian, Black and Other). Although in many cases, more detailed description of ethnicity was available (for example, ‘Black-African’), this was not well reported and general categories such as ‘Black British’ were frequently used. Although collapsing ethnicity into four groups helped to maximise statistical power, it is acknowledged that the inability to look at risk factors among individual ethnic groups is a limitation of this study. In addition, we were unable to look at association between country of birth and stillbirth: at least one previous study reported that the risk of stillbirth for different ethnic groups varied by country of birth (UK vs. non-UK) [8].

Although we had information on smoking, this was only recorded at booking. For those women who are smoking at the start of pregnancy, there is evidence that smoking behaviour tends to fluctuate throughout pregnancy as women attempt to cut down and/or cease smoking [19] and therefore smoking at booking can only be regarded as a proxy for smoking behaviour throughout pregnancy.

### Interpretation

The prevalence of stillbirth in this urban population was 6.2 per 1000 total births. This figure is considerably higher than reported national figures between 2004 and 2012, despite the fact that multiple births were excluded from our study population. It is however, encouraging to note that stillbirth appears to be declining in this population when compared to reported rates of 7.3-8.5 per 1,000 between 2000 and 2010 [20]. The higher prevalence of stillbirth is likely to be in part attributable to the ethnic diversity of this population, and the fact that GSTT is a tertiary referral centre providing specialist care to pregnant women with complex medical conditions.

In this study Black and South Asian ethnicity, obesity, parity and hypertension were all found to be independent risk factors for stillbirth. Although the odds ratios for the association between stillbirth and deprivation, maternal age and diabetes showed trends in line with previous studies (i.e. for advanced maternal age and diabetes the ORs were higher, ORs for lower deprivation were lower), p values were not statistically significant. This may be because the analysis was underpowered to look at these exposures given the rarity of stillbirth. Additionally, most women in this population were considered relatively deprived on a national IMD scale, making it difficult to assess the relationship between deprivation and stillbirth.

In this population, multivariable analysis found that Black and South Asian women had twice the odds of stillbirth compared to White women. This finding is consistent with the results of previous studies reporting a higher risk of stillbirth among non-White women [8]. It has been hypothesised that the increased risk of stillbirth among Black and South Asian women is in part attributable to higher rates of obesity, diabetes, pre-eclampsia and hypertension, high parity and greater deprivation. However, diabetes and deprivation did not have a confounding effect on this relationship in our analysis, and higher odds of stillbirth for all non-White groups remained after parity and hypertension were adjusted for.

Our finding that obesity and ethnicity interact in relation to stillbirth is a novel finding. Specifically, our results suggest that obese South Asian women have a significantly higher risk of stillbirth compared to obese White women. Interestingly, an earlier study conducted using the same population found the effect of obesity on diabetes was significantly stronger in South Asian women compared to White women [21]. Our findings in relation to stillbirth may reflect differences in metabolic susceptibility. They should be considered alongside an increasing body of evidence concluding that the association between obesity and chronic disease (e.g. diabetes) varies by ethnicity. It has been recommended that lower BMI cut-offs should be used to indicate South Asians at higher risk of ill health [22], with a recent suggestion that a similar approach could be extended to Black African and African-Caribbean groups. By using the same BMI obesity threshold for all ethnic groups in our main analysis, it may be that we captured a more ‘extreme’ obese group of South Asian women. To address this concern we ran a sensitivity analysis, the results of which suggest that although the odds of stillbirth associated with obesity are slightly lower for South Asian women when using lower BMI cut-offs, the findings remain significant.

The general increase in stillbirth risk observed in Black women and women from ‘other’ non-White backgrounds may be attributable to ‘social’ or ‘biological’ factors not measured in this study. In terms of social factors, proximal factors such as education, support or knowledge, differences in antenatal screening practice and attitudes to accessing care may be key to explaining the increased risk of stillbirth. In a biological model of causation the intermediaries may be factors such as differences in natural length of gestation [12] and birthweight [23], or higher rates of congenital anomaly due to increased prevalence of diabetes, obesity and consanguinity [24,25].

## Conclusion

This study, conducted in a diverse population with a higher than average stillbirth rate, confirms that ethnicity is an important risk factor for stillbirth. The findings of this study also provide some evidence that the association between obesity and stillbirth differs by ethnic group, with obesity a key risk factor for stillbirth among South Asian women, but less so women from other ethnic backgrounds. This finding is important when clinically assessing high risk pregnancies. Further research is needed into the mechanism by which ethnicity acts to raise the risk of stillbirth in women, particularly in terms of the role of obesity in this relationship.

### Ethical approval

Ethical approval was granted by the London School of Hygiene and Tropical Medicine Ethics committee (ref. 012–326, approved 03 June 2013).

## Additional files

Additional file 1: Table S1. Clinical and socio-demographic factors by stillbirth (≥28 weeks). This table presents the number of births and stillbirths by clinical and socio-demographic factors, limited to births ≥28 weeks. This repeats some of the information in Table 2 using the WHO definition of stillbirth as deaths occurring at ≥28 weeks gestational age. Additional file 2: Table S2. Clinical and socio-demographic factors by ethnic group. This table presents clinical and socio-demographic factors by ethic group, presenting the number of births and stillbirths by ethnic group for all the clinical and socio-demographic factors included in Table 2. Additional file 3: Table S3. Multivariable analysis of clinical and socio-demographic factors associated with stillbirth, using a dummy category for BMI missing (n = 52,403). This table presents the results from a model equivalent to Table 3, including a dummy category for BMI missing. Additional file 4:
**Table S4a.** Multivariable analysis of the association between ethnic group and stillbirth, and obesity and stillbirth, using lower BMI thresholds for South Asian women. This table shows the adjusted odds ratios for the association between ethnic group and stillbirth, and obesity and stillbirth, using ethnicity-specific thresholds for BMI. These results can be compared to those in Table 3. **Table S4b.** Adjusted odds ratios for the association between obesity and stillbirth using lower BMI thresholds for South Asian women, stratified by ethnic group. This table shows odds ratios stratified by ethnic group for the association between obesity and stillbirth using ethnicity-specific thresholds for BMI. These results can be compared to those in Table 4.
