# Supplementary material for: Ethnic variation in stillbirth risk and the role of maternal obesity: analysis of routine data from a London maternity unit
Source: BMC Pregnancy Childbirth. 2014 Dec 7;14:404. doi: 10.1186/s12884-014-0404-0 (PMC4272534; doi:10.1186/s12884-014-0404-0)
Supplement: Additional file 1: Table S1. — Clinical and socio-demographic factors by stillbirth (≥28 weeks). This table presents the number of births and stillbirths by clinical and socio-demographic factors, limited to births ≥28 weeks. This repeats some of the information in Table 2 using the WHO definition of stillbirth as deaths occurring at ≥28 weeks gestational age. [file 12884_2014_404_MOESM1_ESM.docx]

**Table S1. Clinical and socio-demographic factors by stillbirth (≥28 weeks)^1^**

^1^Limited to births at ≥28 weeks, applying WHO definition of stillbirth
