# Supplementary material for: Ethnic variation in stillbirth risk and the role of maternal obesity: analysis of routine data from a London maternity unit
Source: BMC Pregnancy Childbirth. 2014 Dec 7;14:404. doi: 10.1186/s12884-014-0404-0 (PMC4272534; doi:10.1186/s12884-014-0404-0)
Supplement: Additional file 2: Table S2. — Clinical and socio-demographic factors by ethnic group. This table presents clinical and socio-demographic factors by ethic group, presenting the number of births and stillbirths by ethnic group for all the clinical and socio-demographic factors included in Table 2. [file 12884_2014_404_MOESM2_ESM.docx]

**Table S2. Clinical and socio-demographic factors by ethnic group**

*6 months of data for 2012
