# Supplementary material for: Ethnic variation in stillbirth risk and the role of maternal obesity: analysis of routine data from a London maternity unit
Source: BMC Pregnancy Childbirth. 2014 Dec 7;14:404. doi: 10.1186/s12884-014-0404-0 (PMC4272534; doi:10.1186/s12884-014-0404-0)
Supplement: Additional file 3: Table S3. — Multivariable analysis of clinical and socio-demographic factors associated with stillbirth, using a dummy category for BMI missing (n = 52,403). This table presents the results from a model equivalent to Table 3, including a dummy category for BMI missing. [file 12884_2014_404_MOESM3_ESM.docx]

**Table S3. Multivariable analysis of clinical and socio-demographic factors associated with stillbirth, using a dummy category for BMI missing (n=52,403)**

^1^Odds ratios adjusted for all other variables in table
