# Supplementary material for: Ethnic variation in stillbirth risk and the role of maternal obesity: analysis of routine data from a London maternity unit
Source: BMC Pregnancy Childbirth. 2014 Dec 7;14:404. doi: 10.1186/s12884-014-0404-0 (PMC4272534; doi:10.1186/s12884-014-0404-0)
Supplement: Additional file 4: — Table S4a. Multivariable analysis of the association between ethnic group and stillbirth, and obesity and stillbirth, using lower BMI thresholds for South Asian women. This table shows the adjusted odds ratios for the association between ethnic group and stillbirth, and obesity and stillbirth, using ethnicity-specific thresholds for BMI. These results can be compared to those in Table 3. Table S4b. Adjusted odds ratios for the association between obesity and stillbirth using lower BMI thresholds for South Asian women, stratified by ethnic group. This table shows odds ratios stratified by ethnic group for the association between obesity and stillbirth using ethnicity-specific thresholds for BMI. These results can be compared to those in Table 4. [file 12884_2014_404_MOESM4_ESM.docx]

**Table S4a. Multivariable analysis of the association between ethnic group and stillbirth, and obesity and stillbirth, using lower BMI thresholds for South Asian women.**

^1^Odds ratios adjusted for ethnic group, obesity, maternal age (continuous), hypertension and parity

**Table S4b. Adjusted odds ratios for the association between obesity and stillbirth using lower BMI thresholds for South Asian women, stratified by ethnic group**

^1^Odds ratios adjusted for ethnic group, obesity, maternal age (continuous), hypertension and parity
